# Data augmentation method for computer-aided diagnosis using specular reflection

**DOI:** 10.1007/s13534-025-00533-0

**Published:** 2026-01-13

**Authors:** Youmin Shin, Jeonga Seol, Changwoo Lee, Jung Kim, Jinwook Choi, Jinbae Park, Soonwhan Kang, Gyuseon Song, Jung Ho Bae, Young-Gon Kim

**Affiliations:** 1 Department of Transdisciplinary Medicine, Seoul National University Hospital, Seoul, Korea; 2 College of Engineering, Interdisciplinary Program in Bioengineering, Seoul National University, Seoul, Korea; 3 Department of Medical Device Development, Seoul National University College of Medicine, Seoul, Korea; 4 Department of Internal Medicine and Healthcare Research Institute, Healthcare System Gangnam Center, Seoul National University Hospital, Seoul, Korea; 5 Department of Biomedical Engineering, College of Medicine, Seoul National University, Seoul, Korea; 6 Institute of Medical and Biological Engineering, Medical Research Center, Seoul National University, Seoul, Korea; 7 Ainex Corporation, Seoul, Korea; 8 Department of Medicine, Seoul National University College of Medicine, Seoul, Korea; 9 Innovative Medical Technology Research Institute, Seoul National University Hospital, Seoul, Korea

**Keywords:** Specular Reflection, Colonoscopy, Deep learning, Augmentation, Polyp classification

## Abstract

Colorectal cancer (CRC) is a significant global health challenge, emphasizing the importance of effective screening by applying methods like colonoscopy. While advanced imaging technologies, such as narrow-band imaging (NBI), allow real-time optical diagnosis of colon polyps, variations in endoscopist skills and unnecessary polypectomy underscore the need for artificial intelligence applications, particularly deep learning (DL) in computer-aided polyp detection and diagnosis (CADe and CADx). This study developed and investigated a data augmentation technique using specular reflection (SR) to enhance the robustness and performance of DL models tailored explicitly for CADx in colonoscopy. This SR augmentation method included SR generation and inpainting integrated into conventional augmentation techniques. We utilized two DL architectures: a convolutional neural network and a vision transformer. Stress tests, under varying data usage ratios using a dataset of 2,616 NBI images, revealed the robustness of SR augmentation, especially in scenarios with limited training data, highlighting its superiority over other methods. SR augmentation effectively improved model accuracy, particularly in scenarios with limited data, supporting its practical implementation in real-world colonoscopy environments. The findings emphasize the significance of domain-specific data augmentation techniques to support DL application in colonoscopy imaging for more reliable and accurate CADx systems for colon polyps.

**Supplementary Information:**

The online version contains supplementary material available at 10.1007/s13534-025-00533-0.

## Introduction

Colorectal cancer (CRC) is the third most prevalent cancer and has the second-highest mortality rate among all malignancies [1, 2]. Colonoscopy, the gold standard for CRC screening, is pivotal in mitigating its incidence and mortality by effectively identifying and excising premalignant polyps, notably adenomas [3]. However, the prevailing standard practice of endoscopic removal and histological assessment for all detected polyps increases the overall cost of colonoscopy. Furthermore, this approach poses potential risks to patients owing to unnecessary polypectomy procedures, particularly for non-neoplastic polyps such as hyperplastic polyps.

Advanced imaging technologies, such as narrow-band imaging (NBI), allow for real-time optical diagnosis to predict the histologic characteristics of colon polyps during colonoscopy. Endoscopic classification methods, including NBI international colorectal endoscopic [4, 5] classification, differentiate between adenomas and hyperplastic polyps by meticulously characterizing the color, vascular patterns, and surface features [4, 6]. Alternatively, optical diagnosis offers revolutionary strategies in the clinical management of colon polyps, in which low-risk adenomatous polyps are "resected and discarded," while non-adenomatous polyps are "diagnosed and left" in situ.

However, the quality of optical diagnosis, such as the adenoma detection rate, may also vary depending on the endoscopist’s expertise [7]. Currently, artificial intelligence applications are expected to overcome human limitations in colonoscopy. Innovative techniques, such as deep learning (DL), have facilitated computer-aided polyp detection (CADe) and diagnosis (CADx), potentially enhancing the overall quality of colonoscopies and reducing the incidence of unnecessary polypectomies [8].

The efficacy of CADe has been studied in multiple randomized controlled trials, and the results demonstrate a 6.8–9.0% increase in adenoma detection rate with an 11.1–26.1% reduction in the adenoma miss rate compared with the control group [9–13]. CADx has also been studied in prospective single-arm trials; however, its efficacy remains unclear [14, 15]. Moreover, certain instances have revealed the inherent instability or inability of CADx to perform precise characterizations. Therefore, developing a more advanced model is imperative to effectively implement CADx in clinical practice.

Numerous DL investigations with NBI have been conducted to advance CADx systems. These studies have curated substantial datasets, ranging from 2,000 to 70,000 images, for training and have reported accuracy rates of 87–98% [16–22]. For instance, Jin et al. [16] used a neural architecture search based on a convolutional neural network (CNN) trained on 2,150 NBI images (1,100 adenomatous polyps and 1,050 hyperplastic polyps), achieving 86.7% accuracy. Byrne et al. [17] used a CNN model trained on 223 NBI videos comprising 60,089 frames (29% hyperplastic polyps, 53% adenomatous polyps, and 18% normal mucosa with no polyps) and achieved 94% accuracy.

Training on a large dataset (images or frames in the case of videos) is often necessary to build robust, generalizable models [23]. DL approaches for natural image processing adopt various augmentation techniques, such as rotation, flipping, and color jitter, to address the limitations in data collection [24]. However, these techniques may have limited applicability for medical images because of their distinct characteristics, such as high variability and complex structures. Consequently, specialized augmentation methods that consider the unique features of medical images are required to improve DL model performance [25]. Therefore, the medical domain requires augmentation techniques tailored to its specific characteristics; however, few studies have proposed specialized augmentation methods for the medical domain, making it difficult to find methods optimized for endoscopy.

During endoscopy, specular reflections (SR) are inevitable, as the endoscope's light source illuminates the dark intestine’s interior, distinguishing it from examinations in other medical domains. SR often poses a challenge in developing CADx systems because it can hinder the accurate differentiation of polyp characteristics, particularly surface patterns, during colonoscopy. Previous studies have attempted to address this issue by classifying frames with prominent SR as non-informative frames [26, 27]. Although this approach resulted in more filtered images, it necessitated increased data acquisition to achieve high-performance outcomes. Furthermore, these methods failed to produce a reliable system suitable for real colonoscopy scenarios.

This study departs from the conventional approach and introduces an image processing method tailored for colonoscopy images. Instead of categorizing frames with substantial SR as non-informative, our method intentionally generates or inpaints the SR regions. Furthermore, we incorporate SR generation/inpainting as a component of data augmentation designed for CADx using SR. This strategy aims to train a robust algorithm and enable us to compare its effectiveness with natural image augmentation techniques not tailored for CADx purposes. To assess the efficacy and applicability of our approach, we evaluated two types of DL architectures, CNN and transformer.

## Results

### SR generation/inpainting

**Fig. 1:**
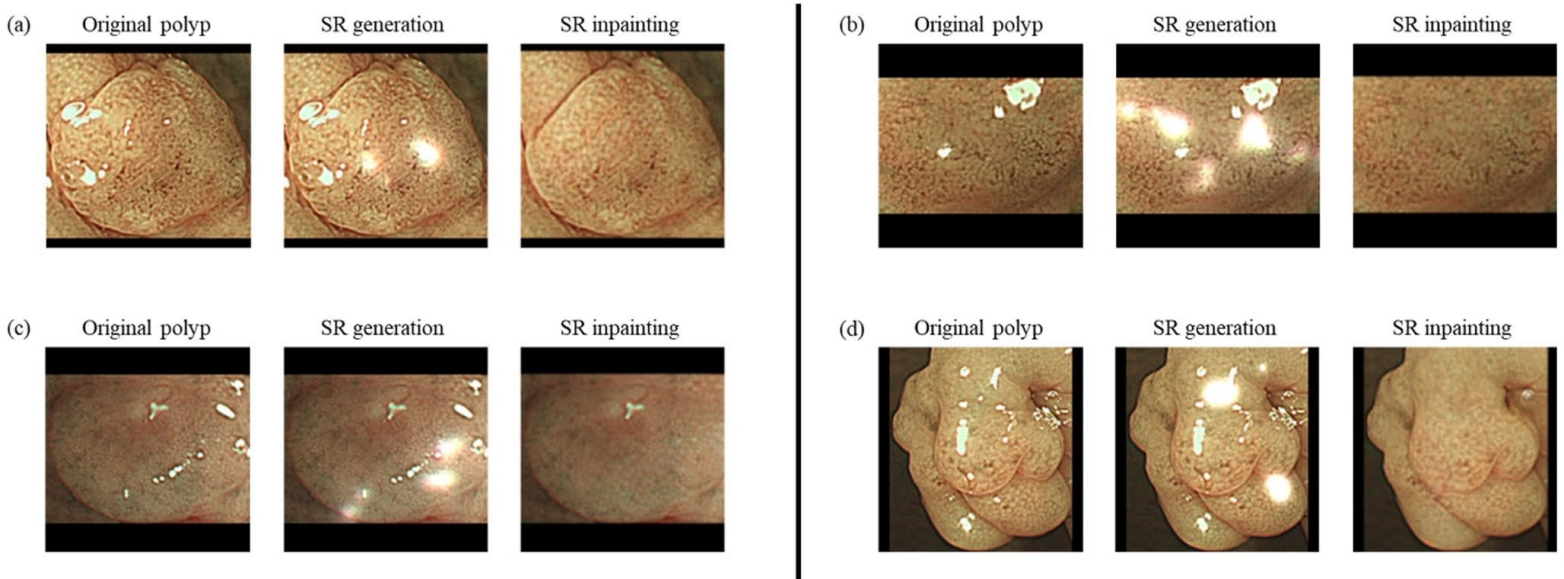
Qualitative examples illustrating SR-based image augmentation. Each row presents the original image (left), the result of synthetic SR generation (center), and the inpainted image. Images (**a**) and (**b**) are adenomatous polyps, while (**c**) and (**d**) are hyperplastic polyps. SR generation simulates specular reflection artifacts by synthetically overlaying bright regions, whereas SR inpainting removes these artifacts using a deep inpainting model. Abbreviation: SR: Specular reflection

Figure 1 illustrates the outcomes of specular reflection (SR) generation (center column) and SR inpainting (right-hand column) in comparison to the original images (left-hand column). Additional examples are provided in Supplementary Fig. 3 for further reference. The depicted polyps were selectively sampled from the adenomatous (AD) and hyperplastic (HP) groups. Through SR generation, the model successfully created images that closely resemble those likely to be encountered in actual colonoscopy scenarios. Similarly, SR inpainting facilitated the generation of images where the polyps appear naturally integrated into the surrounding tissue, enhancing the realism and diagnostic utility of the images.

### SR augmentation for polyp classification

Table 1 details the classification performance of each augmentation method. The baseline for each model was established using standard augmentations common in natural image classification, including rotation, flip, color jitter, Gaussian noise, Gaussian blur, and a combination of all these techniques. The most effective natural augmentation method for each model and input size was then selected as the base augmentation, as listed in Supplementary Table 1.

**Table 1:** Predictive performance for each augmentation

Model	Image size	Augmentation method	AUC	ACC	SEN	SPE	PPV	NPV
ResNet	150 × 150	Baseline	0.895 (0.017)	0.862 (0.016)	0.885 (0.027)	0.800 (0.022)	0.816 (0.017)	0.875 (0.025)
		Baseline + FDA	0.861 (0.006)	0.729 (0.014)	0.790 (0.015)	0.668 (0.025)	0.705 (0.016)	0.761 (0.015)
		Baseline + MixStyle	0.856 (0.012)	0.733 (0.010)	0.787 (0.016)	0.680 (0.014)	0.711 (0.009)	0.761 (0.014)
		Baseline + DDPM	0.939 (0.011)	0.879 (0.012)	0.900 (0.011)	0.958 (0.017)	0.864 (0.012)	0.896 (0.018)
		Baseline + SR_gen	0.925 (0.012)	0.908 (0.017)	0.918 (0.038)	0.878 (0.015)	0.901 (0.012)	0.918 (0.034)
		Baseline + SR_inp	0.893 (0.015)	0.875 (0.019)	0.908 (0.035)	0.851 (0.024)	0.901 (0.015)	0.884 (0.022)
		Baseline + SR_all	**0.938** **(0.013)**	**0.908** **(0.021)**	**0.915** **(0.033)**	**0.892** **(0.023)**	**0.911** **(0.012)**	**0.915** **(0.035)**
	224 × 224	Baseline	0.872 (0.016)	0.868 (0.018)	0.878 (0.037)	0.812 (0.028)	0.838 (0.024)	0.868 (0.016)
		Baseline + FDA	0.909 (0.006)	0.770 (0.008)	0.858 (0.016)	0.682 (0.013)	0.730 (0.008)	0.828 (0.015)
		Baseline + MixStyle	0.887 (0.012)	0.743 (0.016)	0.818 (0.023)	0.668 (0.028)	0.712 (0.016)	0.787 (0.021)
		Baseline + DDPM	0.941 (0.024)	0.875 (0.018)	0.892 (0.013)	0.858 (0.029)	0.863 (0.015)	0.888 (0.021)
		Baseline + SR_gen	0.927 (0.011)	0.912 (0.013)	0.909 (0.025)	0.883 (0.020)	0.887 (0.015)	0.908 (0.021)
		Baseline + SR_inp	0.882 (0.019)	0.881 (0.019)	0.884 (0.038)	0.854 (0.026)	0.894 (0.018)	0.910 (0.025)
		Baseline + SR_all	**0.946** **(0.015)**	**0.918** **(0.015)**	**0.908** **(0.037)**	**0.898** **(0.022)**	**0.904** **(0.015)**	**0.914** **(0.024)**
ViT	150 × 150	Baseline	0.891 (0.015)	0.872 (0.020)	0.874 (0.027)	0.808 (0.025)	0.825 (0.015)	0.874 (0.021)
		Baseline + FDA	0.859 (0.008)	0.732 (0.011)	0.783 (0.020)	0.680 (0.014)	0.710 (0.009)	0.759 (0.017)
		Baseline + MixStyle	0.875 (0.002)	0.719 (0.016)	0.855 (0.017)	0.583 (0.034)	0.673 (0.016)	0.801 (0.017)
		Baseline + DDPM	0.779 (0.021)	0.683 (0.022)	0.642 (0.021)	0.725 (0.027)	0.700 (0.022)	0.669 (0.028)
		Baseline + SR_gen	0.923 (0.014)	0.905 (0.017)	0.915 (0.024)	0.883 (0.017)	0.896 (0.013)	0.913 (0.016)
		Baseline + SR_inp	0.893 (0.011)	0.884 (0.014)	0.893 (0.025)	0.866 (0.028)	0.883 (0.024)	0.912 (0.011)
		Baseline + SR_all	**0.934** **(0.018)**	**0.915** **(0.024)**	**0.916** **(0.028)**	**0.901** **(0.018)**	**0.894** **(0.015)**	**0.915** **(0.025)**
	224 × 224	Baseline	0.893 (0.016)	0.873 (0.013)	0.882 (0.024)	0.810 (0.018)	0.833 (0.014)	0.864 (0.016)
		Baseline + FDA	0.896 (0.011)	0.749 (0.018)	0.842 (0.026)	0.657 (0.030)	0.711 (0.018)	0.806 (0.025)
		Baseline + MixStyle	0.916 (0.009)	0.807 (0.021)	0.850 (0.017)	0.765 (0.029)	0.784 (0.023)	0.836 (0.019)
		Baseline + DDPM	0.922 (0.029)	0.892 (0.025)	0.883 (0.019)	0.900 (0.020)	0.898 (0.018)	0.885 (0.023)
		Baseline + SR_gen	0.914 (0.013)	0.909 (0.012)	0.913 (0.025)	0.882 (0.017)	0.897 (0.011)	0.907 (0.018)
		Baseline + SR_inp	0.895 (0.015)	0.877 (0.015)	0.904 (0.032)	0.859 (0.020)	0.905 (0.019)	0.912 (0.023)
		Baseline + SR_all	**0.925** **(0.015)**	**0.909** **(0.017)**	**0.910** **(0.035)**	**0.904** **(0.020)**	**0.901** **(0.015)**	**0.915** **(0.020)**

AUC: Area under the receiver operating characteristics curve; ACC: accuracy; SEN: sensitivity; SPE: specificity; PPV: positive predictive value; NPV: negative predictive value; FDA: Fourier domain adaptation; DDPM: denoising diffusion probabilistic models

In most cases, regardless of the model or input size, we observed performance improvements when SR augmentation was applied over the baseline augmentation. The most substantial enhancements were noted when SR generation and inpainting were utilized concurrently. While SR inpainting alone sometimes led to minor performance variations, these were not statistically significant (DeLong's test p-value > 0.05). Importantly, combining generation and inpainting yielded a significantly performance improvement than using generation alone. Consequently, the best performance was recorded with an AUC of 0.946 using the ResNet-50 model and a 224 × 224 image size.

### Stress test

**Fig. 2:**
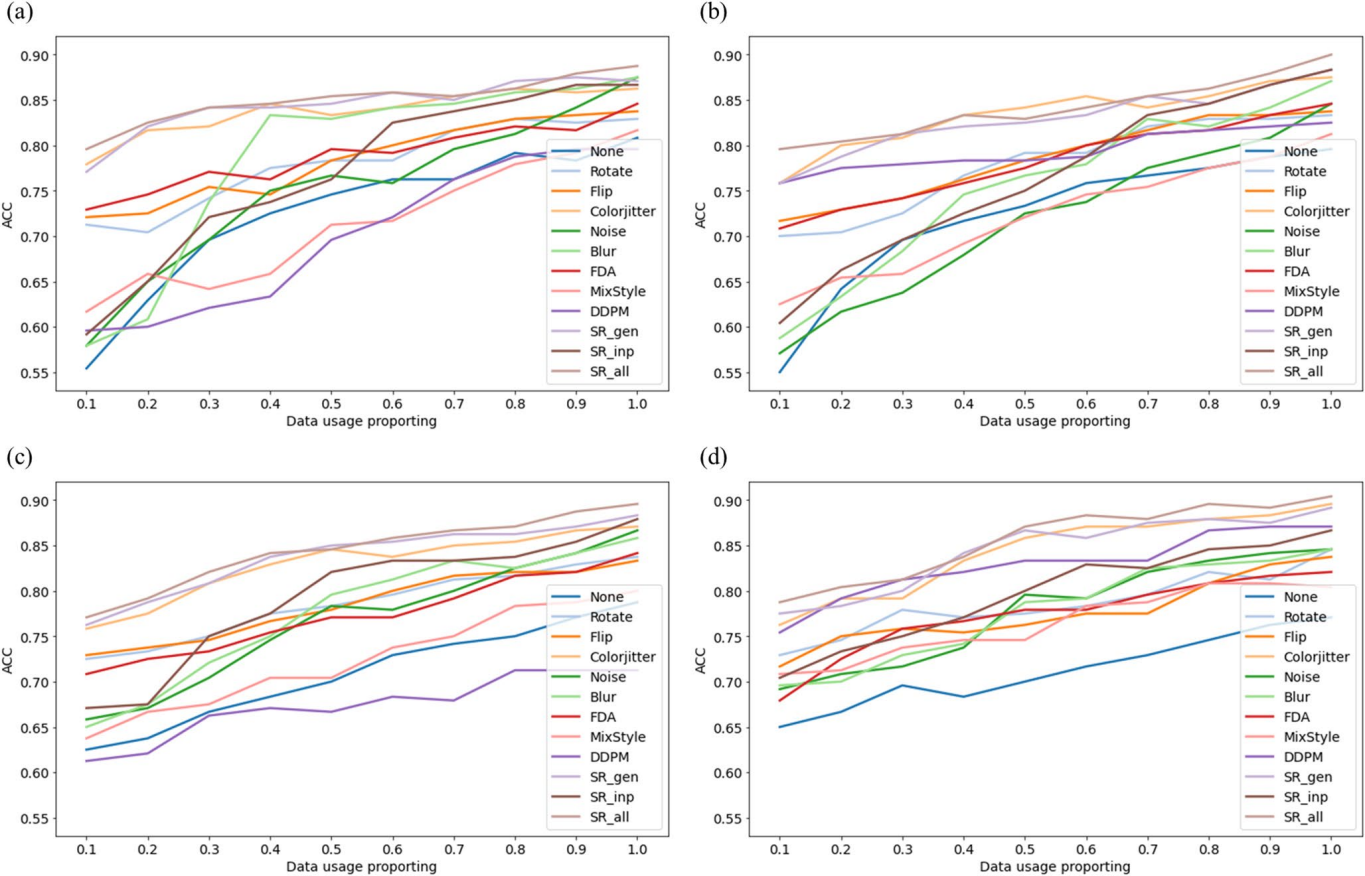
Classification performance under varying SR data usage proportions across model architectures and input data proportion. Each subplot shows model accuracy as a function of the proportion of training images augmented with SR generation and inpainting, varied at 01 intervals (from 0.1 to 1.0). **a** ResNet with 150 × 150 input resolution; **b** ResNet with 224 × 224; **c** ViT with 150 × 150; **d** ViT with 224 × 224. Abbreviations: ACC: Accuracy; FDA: Fourier Domain Adaptation; DDPM: Denoising diffusion probabilistic models; SR: Specular reflection; SR_gen: SR generation; SR_inp: SR inpainting

Figure 2 displays the accuracy of each augmentation method relative to the training data usage ratio. Notably, even with minimal data, the methods involving both SR inpainting and combined SR generation and inpainting achieve high accuracy across various models, and this performance is sustained as data usage increases. Initially, SR inpainting alone exhibits lower performance at low data usage rates, but its accuracy improves significantly with increased data utilization. Combining SR generation and inpainting yields the highest performance gains in most scenarios.

### External validation

We additionally evaluated the impact of SR augmentation on two external datasets: the Swin-Expand dataset and the KUMC dataset. On the Swin-Expand dataset, the SR-augmented model achieved an AUC of 0.846, slightly higher than the baseline model's AUC of 0.834. For the KUMC dataset, the AUC improved from 0.844 to 0.851 at the frame level, and from 0.863 to 0.906 at the video-clip level with SR augmentation. Detailed performance metrics, including accuracy, sensitivity, and specificity, are provided in the supplementary material (Supplementary Table 3).

## Discussion

This study developed and evaluated a novel data augmentation technique using SR specifically designed for colonoscopy image analysis. This approach diverges from traditional methods that typically filter out non-informative frames, offering instead a strategy to classify them via SR inpainting, potentially enhancing the overall data utility.

Visual inspection of both the original images and those modified via SR generation or inpainting revealed that the generated images closely resemble those likely encountered during actual colonoscopy procedures. Additionally, the performance metrics indicated that models augmented with SR techniques outperformed the baseline models. Comparative stress tests against other augmentation methods further underscored the effectiveness of SR augmentation in enhancing the learning process for colonoscopy image analysis.

Table 1 lists the enhanced performance metrics achieved by integrating SR augmentation compared to the baseline, which utilized only natural image augmentation methods. The implementation of SR augmentation afforded significantly higher AUCs and accuracies across all experimental settings, demonstrating its effectiveness regardless of the model architecture or the size of the input images. SR generation contributed to a substantial increase in performance metrics, whereas SR inpainting showed a relatively minor impact. However, combining SR generation and inpainting generally yielded superior results compared to using each method in isolation. Grad-CAM visualizations further supported these findings, revealing that models trained with SR augmentation were able to shift their attention from misleading specular highlights to more clinically relevant polyp structures. This change in attention focus correlates with the observed improvements in classification accuracy (Supplementary Fig. 4).

This finding aligns with the results presented in Fig. 2, which details the outcomes of stress tests conducted while varying the proportion of training data. The tests compare the performances of various augmentation methods. When the dataset is small, color jitter stands out among the natural augmentation methods for its effectiveness, while SR generation shows notable results within the SR augmentation. As the volume of training data increases, except for color jitter, SR inpainting exhibits a more pronounced performance enhancement than other natural augmentation methods. Notably, when the full dataset is utilized, the performance of SR inpainting is comparable to that achieved by SR generation and color jitter.

The performance gains of the two proposed augmentation methods are more significant with SR generation than SR inpainting for the following reasons. In SR inpainting, only one image with an inpainted SR region is used for training. In contrast, SR generation is performed by extracting SR masks from different source images at each epoch, making multiple images available for training. Since more new images can be utilized for training, SR generation offers higher performance than SR inpainting.

While SR augmentation marks a significant improvement, it relies on traditional image processing techniques that do not fully account for the three-dimensional structural relationships within polyps. In future work, we will explore the use of generative models to address this limitation, offering more realistic and spatially coherent augmentations. Moreover, the computational demands of SR inpainting, particularly for real-time applications, highlight the need for optimization. To assess its practical feasibility, we benchmarked the LaMa model on an NVIDIA A6000 GPU with 48 GB memory, observing mean inference times of 13.37 ± 1.65 ms for 150 × 150 images and 16.11 ± 0.66 ms for 224 × 224 images. While acceptable for offline data augmentation during training, these latencies pose challenges for real-time clinical deployment, underscoring the need for further acceleration.

While generative-based augmentation holds significant potential for enhancing dataset diversity, our experiments showed that its performance was slightly inferior to that of SR augmentation methods. This may be caused by the noise introduced during the generation process, which, despite producing visually realistic images, probably disrupted feature learning, particularly in the context of the limited dataset. Furthermore, the synthetic images may have lacked the anatomical precision required for colonoscopy image analysis, highlighting the limitations of generic generative models in addressing domain-specific challenges.

A primary objective in employing data augmentation techniques is to enhance model performance, mainly when the available datasets are small [28]. By artificially expanding the dataset through SR generation and inpainting methods, models can learn more comprehensive and generalized features. This approach improves the robustness and accuracy of the models under constrained data conditions while ensuring that the systems are viable for practical applications, achieving high diagnostic performance with limited real-world data. Notably, the SR augmentation method represents a significant advancement in this area, achieving an accuracy of 0.80 using just 10% of the available data.

In this study, we proposed a specialized data augmentation technique using SR to address the limitations of DL models in analyzing colonoscopy images. While this method marks a significant improvement, it has inherent limitations. One potential concern is that the extracted or augmented SR masks may appear in background areas rather than directly over the polyp itself. However, since SR augmentation is applied within bounding box-guided regions that encompass both the polyp and adjacent mucosa, such occurrences remain clinically plausible. In real-world colonoscopy, SR are commonly observed not only on the polyp surface but also on surrounding tissues and luminal backgrounds. Thus, we consider this a realistic and acceptable artifact that does not compromise the visual authenticity or training effectiveness of the augmented images.

Another limitation stems from the heuristic nature of our SR augmentation pipeline. Specifically, the mask extraction step uses a fixed intensity threshold of 245, which, while effective for detecting bright SR regions, may inadvertently include fragmented high-intensity pixels that are not true specular reflections. This issue can introduce false-positive SR masks. We attempt to mitigate this through post-processing: in SR inpainting, the masked regions are filled based on surrounding tissue textures, and in SR generation, Gaussian filtering and RGB adjustments are applied to smooth sharp artifacts. However, we acknowledge that the absence of morphological operations (e.g., area filtering or shape constraints) limits the precision of SR mask extraction.

In addition, the SR generation process itself is fundamentally heuristic, relying on simple image processing rules without modeling the complex optical properties of light–such as surface curvature or lighting angle–that govern real-world specular reflection. Nonetheless, our goal was not to simulate physically accurate SR phenomena but rather to introduce diverse, clinically plausible perturbations that reflect the variability of actual endoscopic imaging. While no formal clinician rating was conducted, qualitative review by domain experts confirmed that the generated artifacts visually resemble real SR patterns commonly observed during colonoscopy. We have clarified this objective in the manuscript and acknowledged that the visual gap between generated and real SR artifacts remains a technical limitation. This could be addressed in future work through physically grounded SR simulation or structured expert evaluation.

A primary limitation is that SR generation relies on traditional image processing techniques, which involve extracting an SR mask from a source image and applying it to a target image. This approach does not account for the three-dimensional structural relationships within the polyp and its surroundings, potentially leading to SR artifacts appearing in anatomically implausible locations. As a result, such augmented images can sometimes be easily distinguished from real colonoscopy images.

A key strength of our proposed method is its ability to augment datasets effectively in scenarios where data is inherently limited. This is often the case in medical imaging due to privacy restrictions, high annotation costs, and limited access to diverse cases. While our dataset of 2,616 images may appear relatively small, extensive stress tests conducted in this study have consistently demonstrated that the proposed SR augmentation method achieves robust performance improvements across varying dataset sizes. These results indicate that the method saturates its performance effectively and does not require extensive datasets to validate its efficacy. Furthermore, this study highlights the importance of designing augmentation techniques tailored to small data regimes, which aligns with the realities of medical imaging research. By maximizing the utility of existing datasets, the SR augmentation method presents a practical solution for addressing data limitations while maintaining high diagnostic performance.

To overcome these challenges, in future research we will explore the use of deep learning generative models, which are anticipated to yield more realistic augmentations by more accurately representing the spatial dynamics of the scenes. However, deploying such advanced generative models requires larger datasets to train the complex algorithms they entail effectively. Our reliance on smaller datasets constrains our ability to leverage these sophisticated models. Second, SR inpainting, currently performed using the DL-based LaMa model, is significantly more time-consuming than straightforward image processing techniques, hindering its application for real-time inference. Additionally, the efficacy of SR augmentation has not yet been tested in video-based environments, which are critical for endoscopic procedures. Future improvements will optimize the LaMa model to reduce computational demands, potentially enabling real-time processing capabilities and facilitating its deployment in video-based diagnostic systems.

Another important consideration is the potential applicability of the SR augmentation technique to other medical imaging domains. While this study specifically focused on colonoscopy images, the methodology holds promise for generalization to endoscopic images such as gastroscopy and bronchoscopy, where specular reflections are similarly prevalent. Moreover, SR augmentation could potentially be adapted to imaging tasks in radiology, such as ultrasound or CT, by customizing the augmentation to suit modality-specific artifacts. Future research will involve testing the method's efficacy in these broader domains, providing a clearer understanding of its versatility and generalizability.

We further conducted external validation using two independent datasets: KUMC (video-based) and Swin-Expand (still-image-based). The proposed SR augmentation method maintained strong performance on the KUMC dataset. Although video frames generally exhibit lower resolution than still images, we applied a frame-level soft voting strategy that improved patient-level predictions. This confirms the method’s applicability in real-world, video-based CADx settings. On the other hand, results from the Swin-Expand dataset showed that, while AUC remained high, classification accuracy was slightly reduced—likely due to class imbalance.

Despite these challenges, our SR-based data augmentation has demonstrated a significant improvement in model accuracy compared to conventional augmentation methods. Mainly, SR generation exhibited substantial performance enhancement. The strength of this approach is its ability to maintain robust performance, which is especially beneficial in scenarios characterized by limited training data availability. This technique proved valuable in enhancing the performance of DL models for colonoscopy image analysis and aiding the development of more reliable and accurate CADx systems for endoscopic imaging.

## Materials and methods

### Data collection

The endoscopic images were collected from the prospective databases under written informed consent. All colonoscopies were performed using high-definition colonoscopes (EVIS LUCERA CV260SL/CV290SL, Olympus Medical Systems Co., Ltd., Japan) in the Seoul National University Hospital Healthcare System Gangnam Center. The study protocol adhered to the ethical guidelines of the 1975 Helsinki Declaration and its subsequent revisions and was approved by the Seoul National University Hospital Institutional Review Board (number H-2001–083-1095). Additionally, all methods followed the relevant guidelines and regulations, ensuring full compliance with current standards. We obtained 2,616 NBI images from the Seoul National University Hospital Healthcare System Gangnam Center, comprising 1,369 adenomatous polyps (AD) and 1,247 hyperplastic polyps (HP).

### Data split

To ensure a balanced comparison between diagnostic groups, we randomly selected 120 images from each class (approximately 10% of the total dataset) as a hold-out test set, while maintaining a similar distribution of polyp characteristics (Table 2). A chi-square test was conducted to compare the distributions of pathological diagnosis, location, size, and morphology between the training and test sets, confirming no significant differences. For model development, we employed fivefold cross-validation stratified by class to preserve the proportion of AD and HP images in each fold. In each iteration, four folds were used for training and one for validation, enabling robust performance estimation. The test set was strictly held out and not used during training or validation, ensuring an unbiased evaluation of the model’s generalization performance.

**Table 2:** Polyp’s characteristics of the training set and test set

	Training set (N = 2,376)	Test set (N = 240)	*p*-value
Pathologic diagnosis			0.448
AD	1,249 (52.6)	120 (50.0)	
HP	1,127 (47.4)	120 (50.0)	
Location			0.783
Left side colon	1,309 (55.1)	130 (54.2)	
Right side colon	1,067 (44.9)	110 (45.8)	
Size			0.753
Diminutive (≤ 0.5 cm)	1,928 (81.1)	197 (82.1)	
Small (0.6 ~ 0.9 cm)	382 (16.1)	35 (14.6)	
Large (≥ 1.0 cm)	66 (2.8)	8 (3.3)	
Morphology			0.849
Protruded	571 (24.0)	59 (24.6)	
Flat	1,805 (76.0)	181 (75.4)	

AD: Adenomatous polyp; HP: Hyperplastic polyp

Values in parentheses represent the percentage of each category within the corresponding dataset (training set: N = 2,376, test set: N = 240)

### Preprocessing

In the analysis of still cuts from original colonoscopy images, the bounding box marked by the colonoscopist, indicating the polyp's position, was used. Given the varying width-to-height ratios of these bounding boxes, directly resizing the cropped polyp areas could significantly distort the original polyp pattern. To prevent this and preserve the integrity of the original pattern, padding was applied to the image's top, bottom, left, and right sides before resizing.

### Base augmentation

The most used methods—rotation, flipping, color jitter, Gaussian noise, and Gaussian blur—were selected for base augmentation. Additionally, coordination noise was introduced, which involves cropping patches around the center point of the bounding box. This process ensures a more diverse set of images is used as input. Coordination noise is defined as a random variable drawn from a uniform distribution ranging from –α to α (where α is a positive number). The dimensions of the cropped polyp image are then adjusted by multiplying them by the original bounding box's width and height.

### SR augmentation

**Fig. 3:**
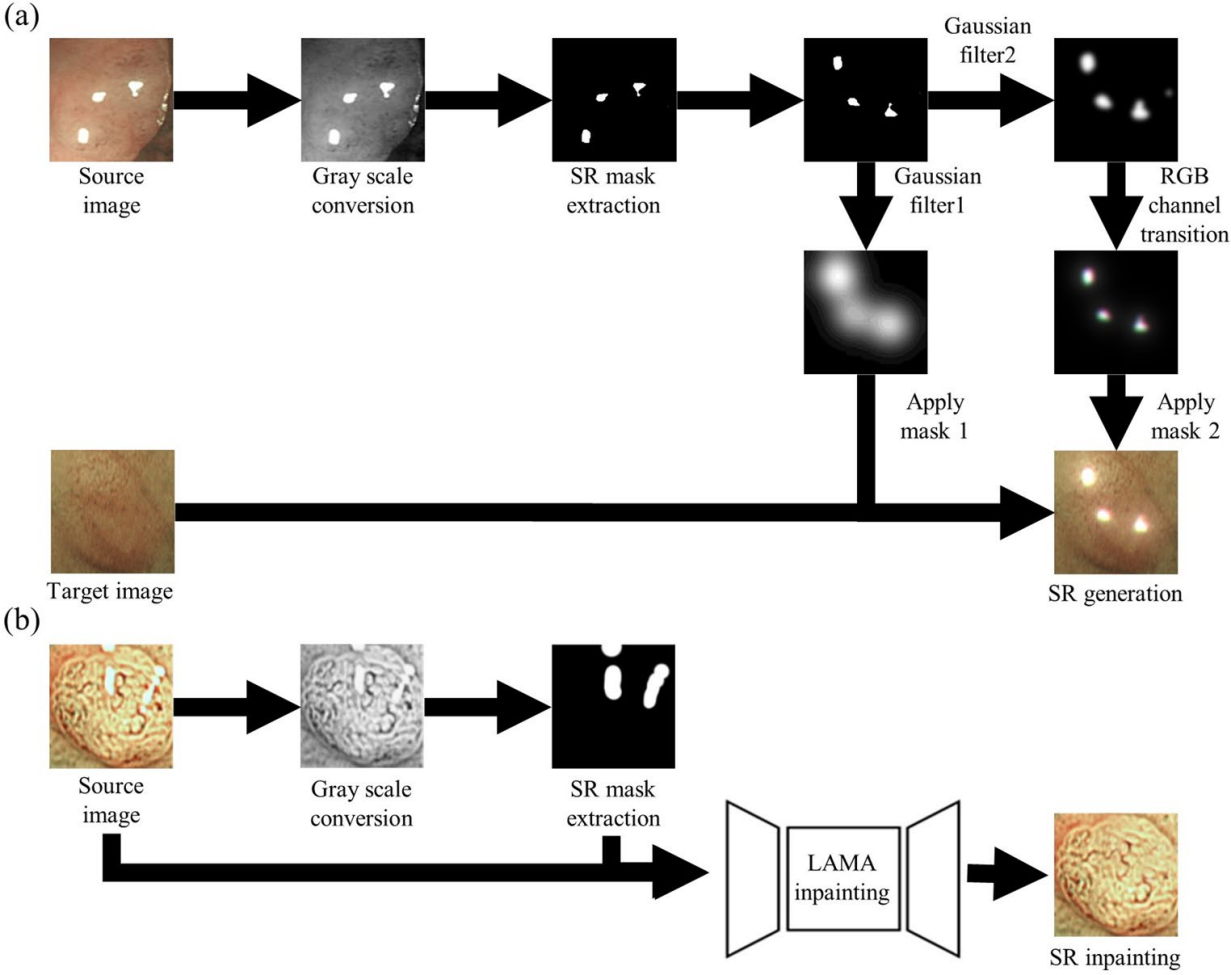
SR augmentation flow chart; **a** SR generation; **b** SR inpainting. Abbreviations: SR: Specular reflection; SR_gen: SR generation; SR_inp: SR inpainting


In the case of SR generation, to simulate realistic SR, we extract SR masks by converting polyp images to grayscale and applying a binary threshold (> 245). The SR mask is then refined using two Gaussian filters with different kernel sizes (*k*₁ = 5 and *k*₂ = 0) to create both smooth highlights and localized color spreading effects. We further enhance diversity by rotating or flipping the mask and applying RGB perturbation to simulate color blotches typically seen around SR regions. This processed SR mask is overlaid onto a randomly selected target image from the same class to create a synthetic SR-augmented image. The pseudocode for the SR generation process is summarized in Algorithm 1, and the visual outcome is shown in Fig. 3a.

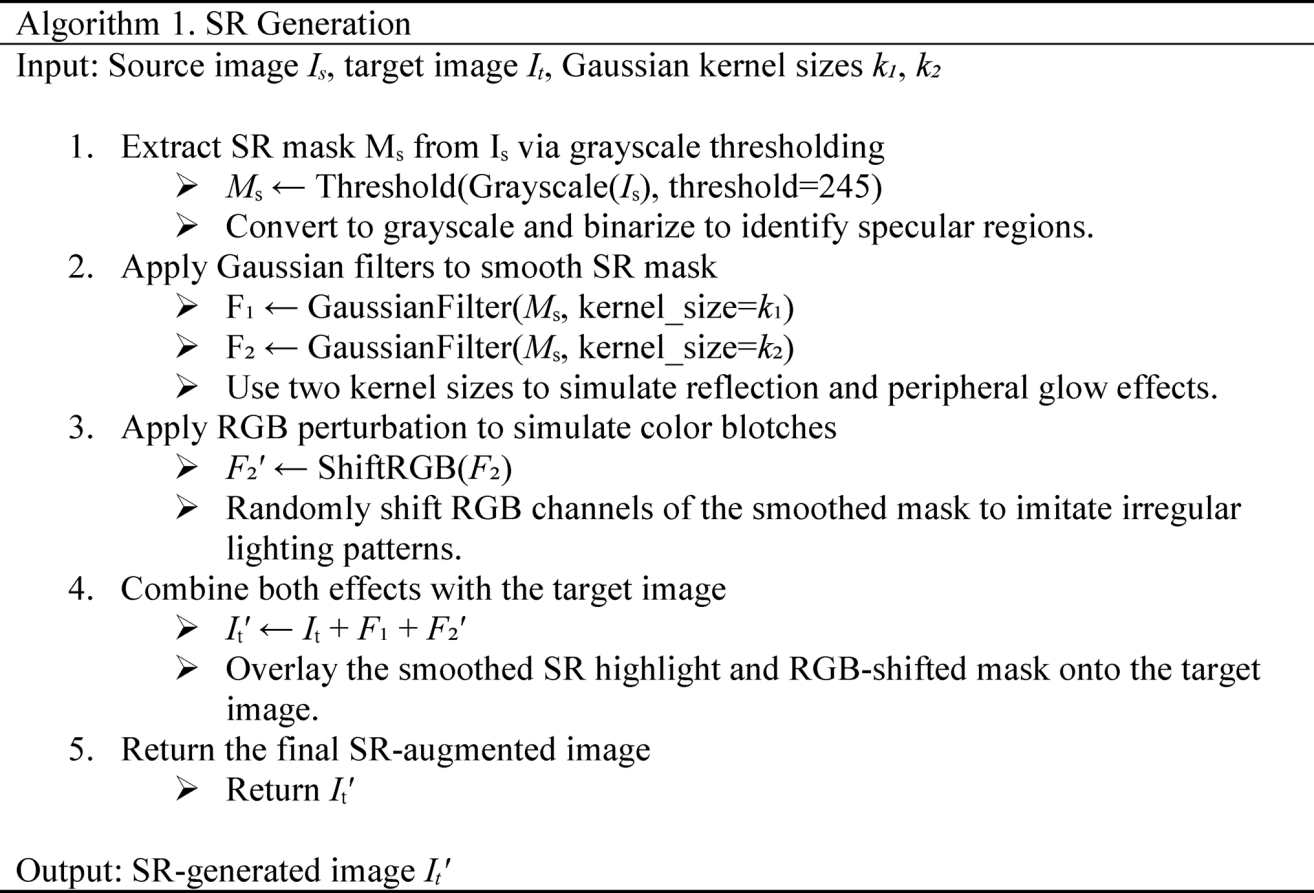


For the SR inpainting, we utilized Large Mask Inpainting (LaMa) [29], a method specifically designed to address challenges such as large missing areas, complex geometric structures, and high-resolution images, which are common in image inpainting tasks. The primary issue in traditional inpainting methods is the inadequate receptive field in the network and loss function, which limits their ability to restore large and intricate missing regions effectively. LaMa overcomes these challenges through three key innovations. First, it employs Fast Fourier Convolutions (FFC), which provide an image-wide receptive field by splitting the convolutional process into local and global branches. This enables the model to blend spatial details efficiently with global structural coherence. Second, LaMa incorporates a high receptive field perceptual loss, ensuring the consistency of global structures by leveraging Fourier or dilated convolutions to expand the receptive field. Third, it adopts an aggressive mask generation strategy, using wide and large masks during training to fully exploit the high receptive field capabilities of FFC and the perceptual loss. With these architectural features LaMa is particularly well-suited for inpainting the SR regions of polyps, which are characterized by intricate gyrus patterns and complex visual structures. By utilizing a network architecture with a wide receptive field, LaMa effectively restores the visual coherence of the augmented regions.

The SR inpainting process is described in Algorithm 2. First, the SR mask ${M}_{S}$ is extracted by applying grayscale conversion and high-value thresholding to the source image ${I}_{S}$. This mask highlights the bright specular regions that disrupt structural continuity. To smooth the boundaries and better guide the inpainting model, the mask is dilated using a rectangular kernel (e.g., 10 × 10). The unaffected portion of the image $\widetilde{{I}_{S}}$ is then isolated via a bitwise masking operation. The inpainting task is performed by LaMa, a high-resolution inpainting model that leverages Fast Fourier Convolutions and high receptive field perceptual loss. This enables the network to restore complex tissue structures while maintaining global consistency. The final output ${I}_{Inp}$ contains realistically reconstructed SR regions, improving the structural integrity of the image.

All hyperparameters used in the SR augmentation processes—including the binary threshold value for SR mask extraction (> 245), Gaussian filter kernel sizes (k₁ = 5, k₂ = 0) for SR generation, and the dilation kernel size (k = 10) for SR inpainting—were selected empirically. We systematically tested multiple candidate values and visually inspected the resulting SR masks and augmented images.


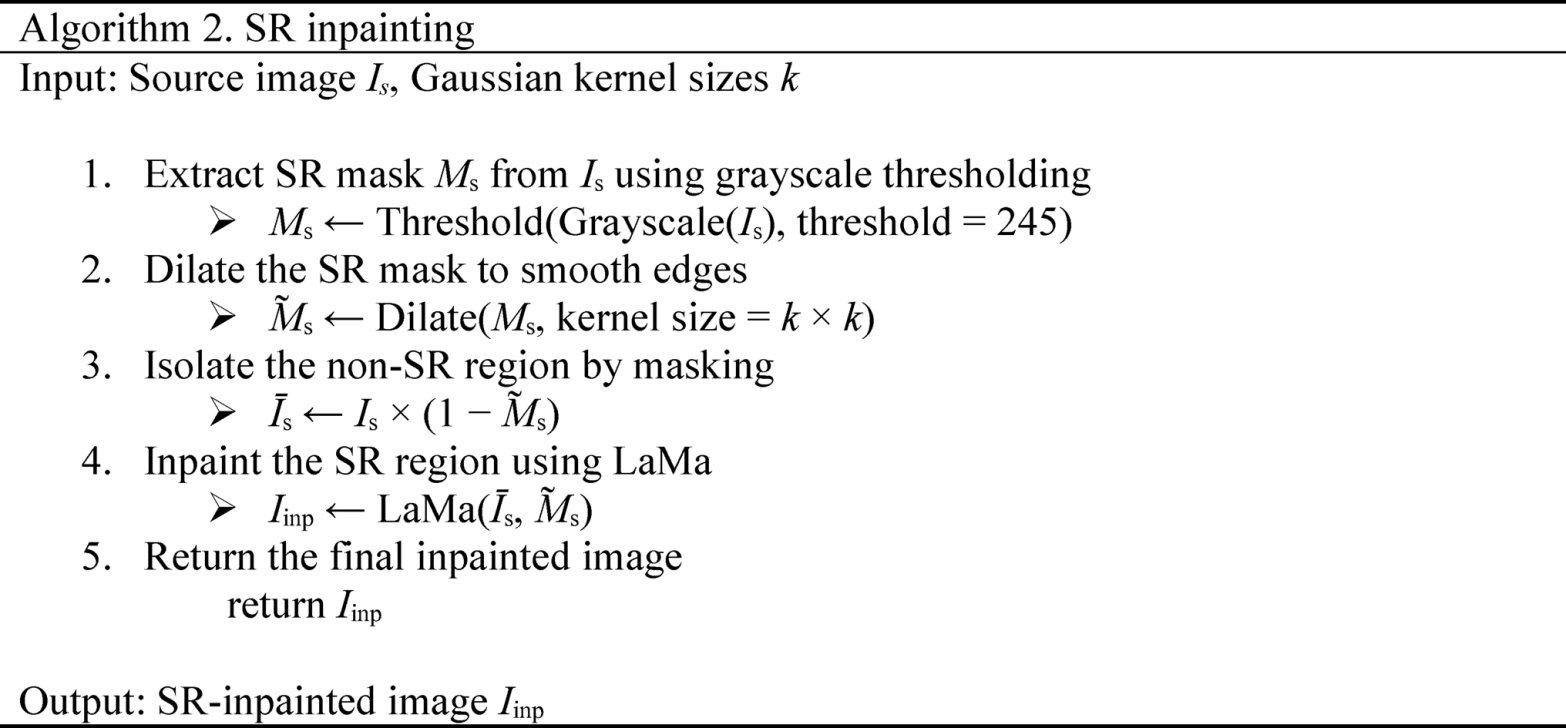


### Comparative augmentation strategies

We employed two recent augmentation strategies commonly used in medical image analysis—Fourier Domain Adaptation (FDA) [30, 31] and MixStyle [32]—to enhance model robustness against domain shifts. FDA operates in the image space by replacing the low-frequency components of a source image with those from a target image, effectively simulating inter-domain variations such as lighting and color tone. In contrast, MixStyle performs style mixing in the feature space by interpolating the channel-wise mean and standard deviation between feature maps of different samples, thus promoting style-invariant feature representations during training. While FDA introduces variability at the pixel level, MixStyle encourages the model to learn more generalizable patterns in the representation space.

In addition, Generative augmentation was utilized to enhance dataset diversity by creating realistic synthetic polyp images that replicate real-world characteristics. These synthetic images were incorporated into the training set of the classification model. Two generative approaches, WGAN [33] (Supplementary Fig. 1) and diffusion-based method, DDPM [34]] (Supplementary Fig. 2), were applied. However, due to the suboptimal performance of GANs on the available dataset, the main analysis focused exclusively on DDPM-generated synthetic datasets. To determine the optimal ratio of synthetic to real images in the classification training data, we conducted experiments, with the results summarized in Supplementary Table 2.

### Deep learning

Images with 150 × 150 and 224 × 224 pixels were used to assess the efficacy of the augmentation techniques with different input image sizes. Additionally, ResNet [35] and vision transformer (ViT) [36] were employed to determine the achievability of robust higher performance with augmentation regardless of the DL model.

ResNet is a DL architecture designed using CNN layers. We introduced a residual block that adds the block's input value to the output value to address vanishing gradients during DL training. This allowed the network to be configured and trained to greater depths, resulting in excellent performance across various computer vision tasks.

ViT was designed to extend the transformer architecture, which was revolutionary in natural language processing, to computer vision. The idea was to split the image into smaller patches and use them as input to the transformer model. This structure allows ViT to effectively perform various computer vision tasks by capturing the relationship between patches in the image through self-attention operation.

### Stress test

Stress tests were conducted to ensure that the data augmentation method is effective even for small training data, which is the primary purpose of data augmentation. We set the overall ratio of learning data to 100%, increased the ratio of training data from 10 to 100% in increments of 10%, and compared the test performance of the trained model against training data selected through random sampling by that percentage.

### External validation

To evaluate the generalizability and clinical relevance of the proposed SR augmentation method, we conducted additional validation experiments using two independent datasets: KUMC [37] and Swin-Expand [38]. The KUMC dataset comprises colonoscopy video frames, enabling validation in a video-based setting. As video frames typically exhibit lower image quality compared to still images, we employed a temporal voting strategy that aggregates predictions across consecutive frames to improve robustness and reduce prediction noise.

### Experimental setting

All implementations were performed using PyTorch v1.8.0 (39), and all experiments were conducted using a cosine annealing scheduler with an early-stopping method. All models were trained and evaluated on a single NVIDIA A6000 GPU with 48 GB memory.

## Supplementary Information

Below is the link to the electronic supplementary material.

Supplementary file1 (DOCX 8405 kb)

## Data Availability

The data, analytic methods, and study materials used in this study will be made available to other researchers upon proper request.
