# Supplementary material for: Data augmentation method for computer-aided diagnosis using specular reflection
Source: Biomed Eng Lett. 2026 Jan 13;16(2):493–505. doi: 10.1007/s13534-025-00533-0 (PMC13013768; doi:10.1007/s13534-025-00533-0)
Supplement: Supplementary file 1 — Supplementary file1 (DOCX 8405 kb) [file 13534_2025_533_MOESM1_ESM.docx]

Supplementary Material

# Supplementary Figures

Supplementary Figure 1. Polyps generated using diffusion models.


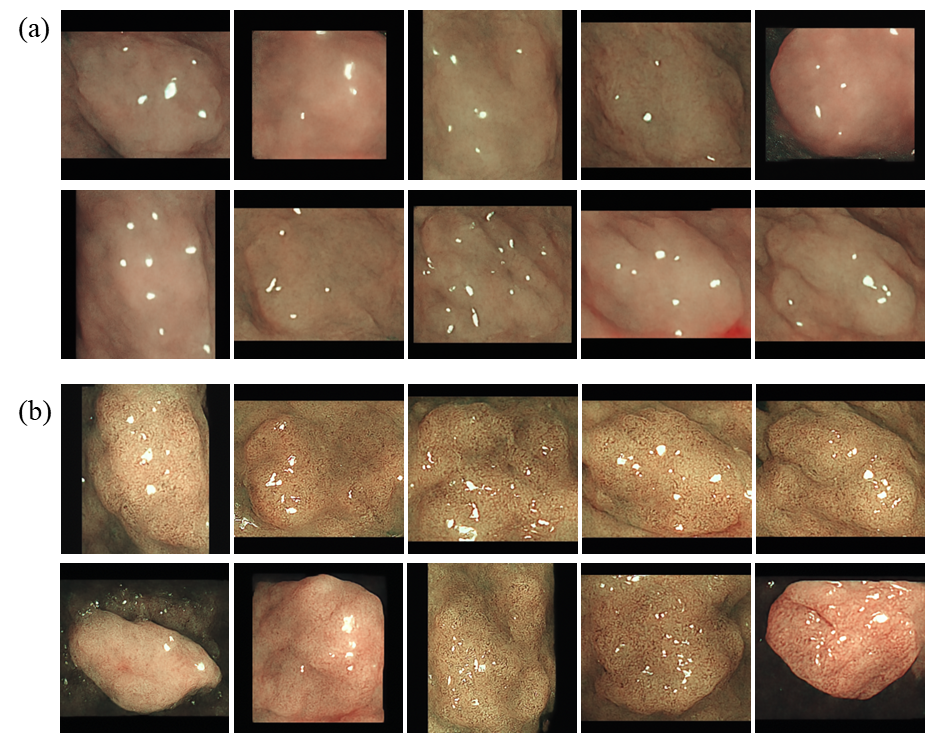


# (a) Generated HP polyp images, (b) Generated AD polyp images.

*AD: Adenomatous polyp; HP: Hyperplastic polyp*

Supplementary Figure 2. Polyps generated using GAN models.


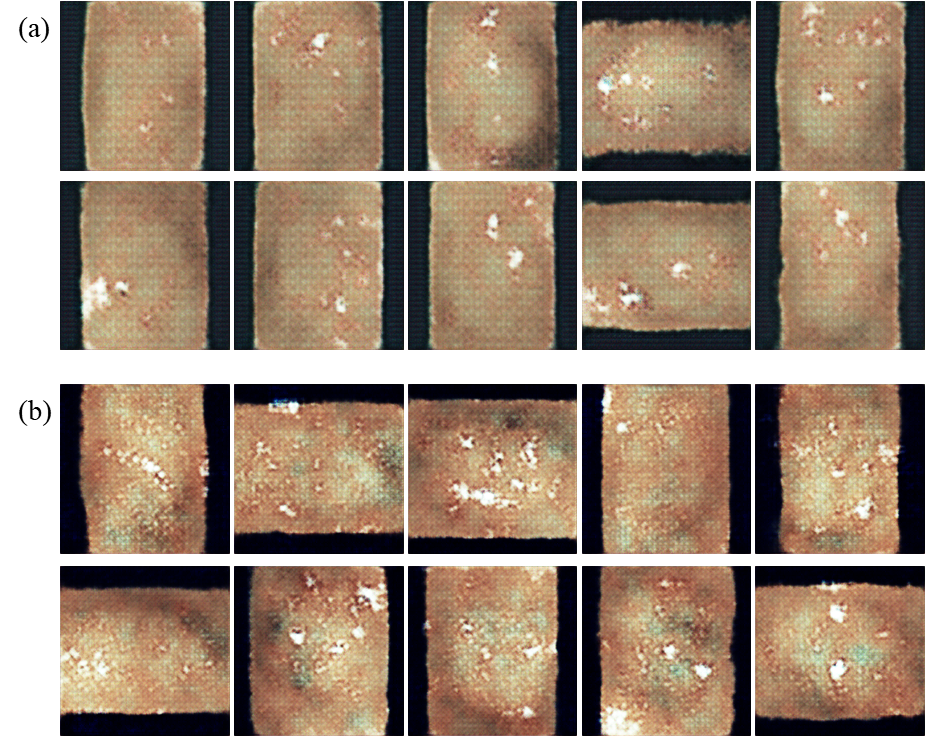


# (a) Generated HP polyp images, (b) Generated AD polyp images.

AD: Adenomatous polyp; HP: Hyperplastic polyp

Supplementary Figure 3. SR generation (center column) and SR inpainting (right column) results


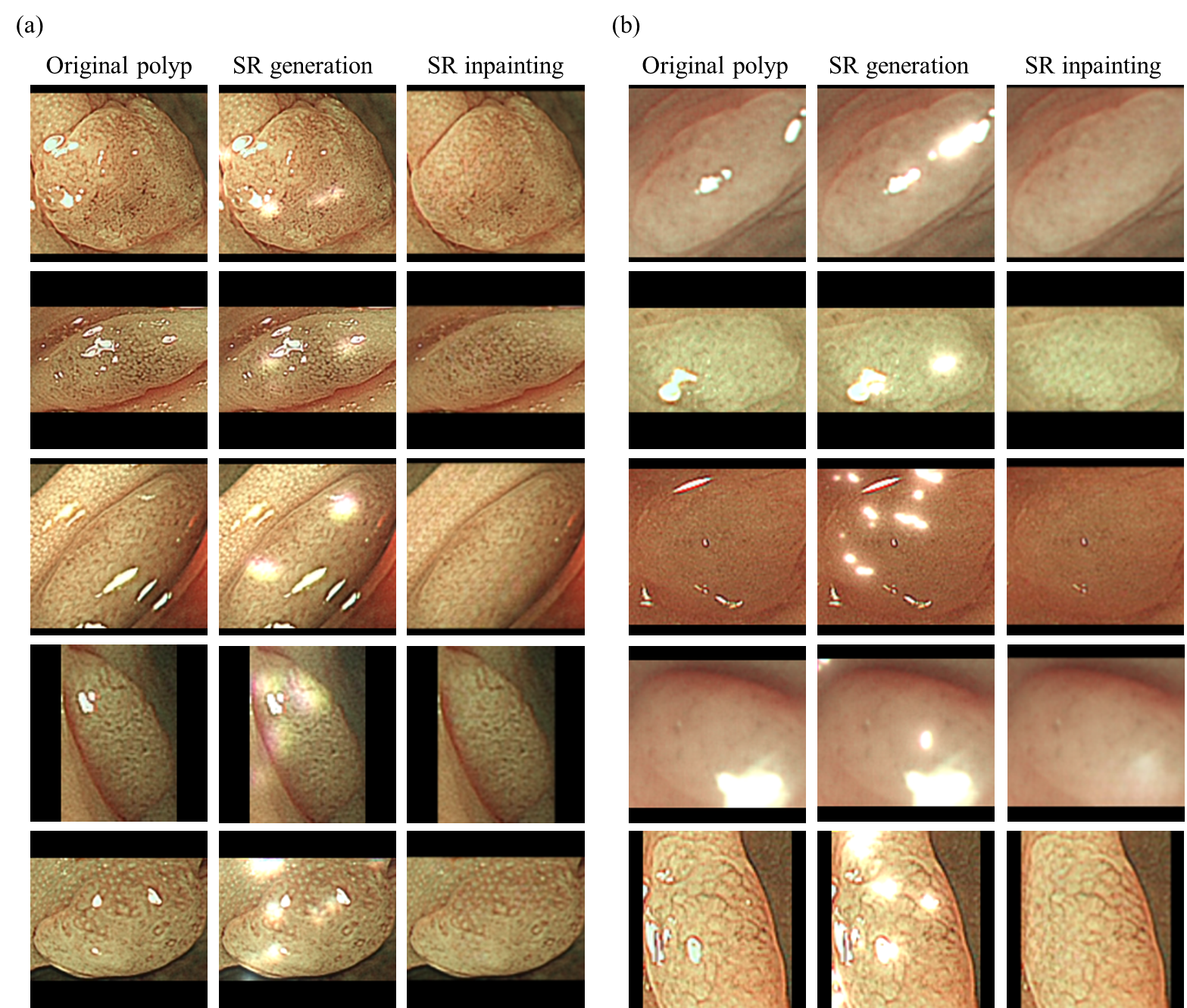


(a) adenomatous polyps; (b) hyperplastic polyps

SR: Specular reflection; SR_gen: SR generation; SR_inp: SR inpainting

Supplementary Figure 4. Grad-CAM visualizations comparing baseline and SR-augmented models, highlighting shifts in attention from specular reflection artifacts to diagnostically relevant polyp regions.

**
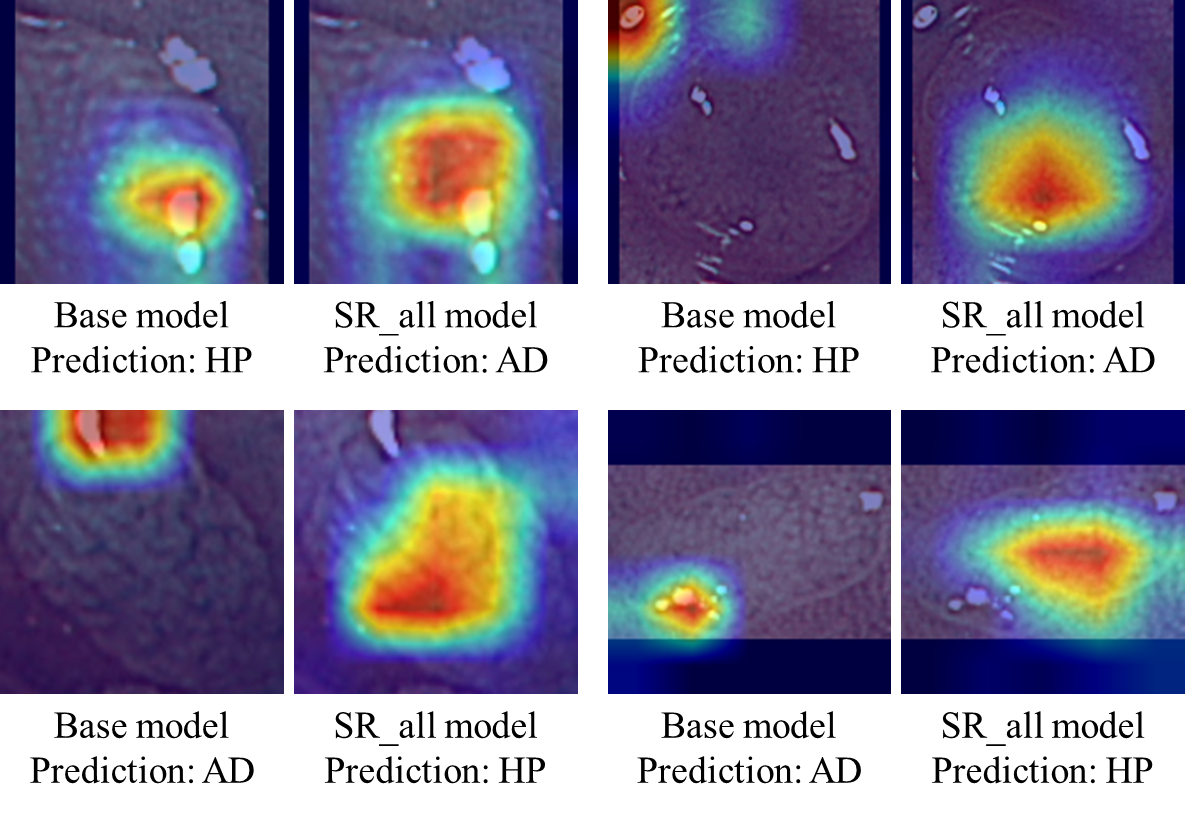
**

# Supplementary Tables

Supplementary Table 1. Predictive performances for conventional augmentation methods

| **Model** | **Image size** | **Augmentation method** | **ACC** |
| --- | --- | --- | --- |
| ResNet | 150 × 150 | None | 0.808 |
|  |  | Rotation | 0.829 |
|  |  | Flip | 0.838 |
|  |  | Color jitter | 0.863 |
|  |  | Gaussian noise | 0.871 |
|  |  | Gaussian blur | 0.875 |
|  |  | **All** | **0.900** |
|  | 224 × 224 | None | 0.796 |
|  |  | Rotation | 0.833 |
|  |  | Flip | 0.838 |
|  |  | Color jitter | 0.875 |
|  |  | Gaussian noise | 0.846 |
|  |  | Gaussian blur | 0.871 |
|  |  | **All** | **0.896** |
| ViT | 150 × 150 | None | 0.788 |
|  |  | Rotation | 0.838 |
|  |  | Flip | 0.833 |
|  |  | Color jitter | 0.871 |
|  |  | Gaussian noise | 0.867 |
|  |  | Gaussian blur | 0.858 |
|  |  | **All** | **0.904** |
|  | 224 × 224 | None | 0.771 |
|  |  | Rotation | 0.842 |
|  |  | Flip | 0.838 |
|  |  | Color jitter | 0.896 |
|  |  | Gaussian noise | 0.842 |
|  |  | Gaussian blur | 0.846 |
|  |  | **All** | **0.908** |

ACC: Accuracy

Supplementary Table 2. Predictive performance based on augmented image ratios

| **Model** | **Image size** | **Augmentation method** | **Generative**  **ratio (%)** | **ACC** |
| --- | --- | --- | --- | --- |
| ResNet | 224 × 224 | Baseline  + DDPM | 10 | 0.842 |
|  |  |  | 30 | 0.871 |
|  |  |  | 50 | 0.875 |
|  |  |  | 100 | 0.867 |

ACC: Accuracy; DDPM: Denoising diffusion probabilistic models

Supplementary Table 3. Comparison of diagnostic performance with and without SR augmentation on external datasets.

| Dataset | Level | Model | AUC | Accuracy (%) | Sensitivity (%) | Specificity (%) |
| --- | --- | --- | --- | --- | --- | --- |
| Swin-Expand | Image | Baseline | 0.834 | 69.7 | 63.5 | 92.5 |
|  |  | SR-Augmented | 0.846 | 68.6 | 62.2 | 92.5 |
| KUMC | Frame | Baseline | 0.844 | 78.3 | 75 | 83.4 |
|  |  | SR-Augmented | 0.851 | 78.8 | 76.3 | 82.7 |
|  | Video | Baseline | 0.863 | 86.4 | 84.6 | 88.9 |
|  |  | SR-Augmented | 0.906 | 90.9 | 92.3 | 88.9 |

AUC: Area under the receiver operating characteristic curve; ACC: Accuracy
